# Stricture of the Œsophagus

**Published:** 1867-11

**Authors:** J. A. Bowers


### SELECTIONS.

*Stricture of the Œsophagus.* Read before the Giles County Medical Association, April 1st, 1867. By Dr. J. A. Bowers, and requested for publication by the Society.

On the 27th of December, 1865, I was requested to see Miss L. M., aged 20 years. I found her in bed, extremely emaciated—pulse quick and feeble—bowels constipated, and mentally much depressed.

According to her own statement and that of her family, she enjoyed perfect health until the 15th of April previous, at which time she swallowed through mistake, one or two fluid ounces of strong lye. The fluid was immediately ejected, followed by severe pain and inflammation of the fauces, œsophagus and stomach, which raged for several days, notwithstanding the active antiphlogistics resorted to by her attending physician. From the date of the injury to the time I first saw her, which was a little more than seven months, she was unable to swallow solid food of any kind, and so great was the difficulty in deglutition, she was compelled to take an ordinary glassful of water in several distinct portions. Her diet consisted of milk and thin soup, requiring about three hours at each regular meal to pass a sufficient quantity to sustain life.

As I was not prepared on my first visit to make a thorough examination of the œsophagus, and feeling convinced that I had organic stricture to contend with, I prescribed Calomel grs. iii, to be taken every three hours until slight ptyalism was produced. As a nourishment, I recommended enemata of milk and beef tea.

I called again on the 28th and found my patient in the same condition as on the day previous. Being now armed with gum catheters of various sizes, I determined to explore the œsophagus and learn the true nature of the case. Placing my patient in a chair, with her head resting on my breast so as to bring the mouth on a line with the fauces, I attempted to pass gum catheter No 10, but it met with firm resistance at a point about eight inches from the incisor teeth, and would go no further—at least with such force as I thought it prudent to employ. I then introduced and passed Nos. 6 and 8, the latter, however, not without some force.

Three days later, I passed Nos. 10 and 12 with very slight force—the patient by this time being, to some extent, under the influence of mercury. I continued to pass bougie No. 12 every third day, gradually enlarging the instrument with wax, until the stricture was entirely overcome, which was accomplished within six weeks of the first operation.

As I could not yet discover any improvement in the case so far as deglutition was concerned, I concluded there must be one or more strictures beyond the reach of the gum bougie. I accordingly procured the red mulberry root of proper size, and length sufficient to reach the stomach ; and being rendered perfectly smooth by a coat of wax, I attempted to pass it to the stomach, but it was arrested at a point about three inches from the cardiac orifice. Avoiding, as I did in the first stricture, undue force on the instrument, I pressed on it sufficiently to ascertain the extent and resistence of the stricture, and size of the orifice, which was plainly impressed upon the instrument, thus enabling me to determine the size of instrument necessary to be employed with benefit.

On the following day I employed mulberry bougie size of No. 12 gum bougie, though not without force and some pain to the patient, the stricture being firm and cartilaginous.

I continued my operations every four days for two weeks—then every third day, and finally every day, gradually increasing the size of the bougie with wax until every vestige of the stricture was obliterated, and the patient enabled to swallow any kind of diet with ease.

Mercury was freely used in this case, and ptyalism persisted in during the first eight weeks of treatment. Five months from the date of my first visit she was discharged cured.

My experience in this case has led me to the opinion that many organic strictures of the œsophagus, which at first appear incurable, will be found to yield to careful and protracted dilatation by the bougie—without the use of caustic as recommended by authors on the subject. The use of the bougie is in no way dangerous, if due care be taken—and I

found the patient's objections to the instrument of short duration.

During the treatment, I found dilatation to occur very gradually; and frequently there appeared to be a re-contraction, which rendered it necessary to return to a smaller size of instrument.

At the date of the injury my patient weighed 180 pounds —seven months subsequent, at which time I first saw her, she weighed 105 pounds—she now weighs 182 pounds, and is apparently in the enjoyment of perfect health.—*Nashville Journal of Medicine & Surgery.*

